# The Positive Regulatory Roles of the TIFY10 Proteins in Plant Responses to Alkaline Stress

**DOI:** 10.1371/journal.pone.0111984

**Published:** 2014-11-06

**Authors:** Dan Zhu, Rongtian Li, Xin Liu, Mingzhe Sun, Jing Wu, Ning Zhang, Yanming Zhu

**Affiliations:** 1 College of Life Science, Qingdao Agricultural University, Qingdao, P.R. China; 2 Key Laboratory of Molecular Biology, College of Heilongjiang Province, Heilongjiang University, Harbin, P.R. China; 3 Plant Bioengineering Laboratory, Northeast Agricultural University, Harbin, P.R. China; National Taiwan University, Taiwan

## Abstract

The TIFY family is a novel plant-specific protein family, and is characterized by a conserved TIFY motif (TIFF/YXG). Our previous studies indicated the potential roles of TIFY10/11 proteins in plant responses to alkaline stress. In the current study, we focused on the regulatory roles and possible physiological and molecular basis of the TIFY10 proteins in plant responses to alkaline stress. We demonstrated the positive function of TIFY10s in alkaline responses by using the *AtTIFY10a* and *AtTIFY10b* knockout Arabidopsis, as evidenced by the relatively lower germination rates of *attify10a* and *attify10b* mutant seeds under alkaline stress. We also revealed that ectopic expression of *GsTIFY10a* in *Medicago sativa* promoted plant growth, and increased the NADP-ME activity, citric acid content and free proline content but decreased the MDA content of transgenic plants under alkaline stress. Furthermore, expression levels of the stress responsive genes including *NADP-ME*, *CS*, *H^+^-ppase* and *P5CS* were also up-regulated in *GsTIFY10a* transgenic plants under alkaline stress. Interestingly, *GsTIFY10a* overexpression increased the jasmonate content of the transgenic alfalfa. In addition, we showed that neither GsTIFY10a nor GsTIFY10e exhibited transcriptional activity in yeast cells. However, through Y2H and BiFc assays, we demonstrated that GsTIFY10a, not GsTIFY10e, could form homodimers in yeast cells and in living plant cells. As expected, we also demonstrated that GsTIFY10a and GsTIFY10e could heterodimerize with each other in both yeast and plant cells. Taken together, our results provided direct evidence supporting the positive regulatory roles of the TIFY10 proteins in plant responses to alkaline stress.

## Introduction

Salt-alkaline stress is one of the most severe environmental challenges and affects all aspects of plant physiological and metabolic processes [1], [2]. Compared with neutral salt stress, soil alkalization leads to high pH stress, poor fertility, dispersed physical property and low water content, and thereby causes much stronger inhibition of plant growth and development [3]–[5]. In recent years, research on plant responses to salt stress have identified the molecular basis of stress signal transduction pathways and salt tolerance mechanisms, and have been at the forefront of plant stress biology [6]. Unfortunately, until now, little attention has been paid on the molecular mechanisms of plant responses to alkaline stress [7], [8].

Alfalfa (*Medicago sativa L.*) is an important worldwide leguminous forage crop and distributes over a wide range of climatic conditions [9], [10]. It has become one of the most important plants due to its high productivity, high feed value and potential roles in soil improvement and soil conservation [11], [12]. However, alfalfa yield and symbiotic nitrogen-fixation capacity were severely restricted by adverse environmental stresses, especially soil salinity and alkalinity [10], [13]. With the global climate change and the global shrinkage of arable lands, a grimmer reality of soil salinity and alkalinity is painted. Therefore, it is of fundamental importance to explore salt/alkaline-tolerant alfalfa through rational breeding and genetic engineering strategies.

The TIFY family, a novel plant-specific protein family, is characterized by a conserved TIFY motif (TIFF/YXG), and comprises 18 members in *Arabidopsis* and 20 members in rice [14], [15]. It has been well suggested that TIFY genes play important roles in the jasmonate (JA) signaling pathway [16], [17], plant growth and development[18]–[21], and pathogen responses [22]–[24]. For example, in *Arabidopsis*, TIFY genes are suggested to negatively regulate the key transcriptional activator of JA responses [25], such as MYC2 [26], MYC3, MYC4 [26], [27], MYB21, MYB24 [28], bHLH017 and bHLH003 [29]. Furthermore, *AtTIFY4a* and *AtTIFY4b* regulate lamina size and curvature [30], whereas *AtTIFY1* plays a role in petiole and hypocotyl elongation [31]. Recent research in tomato and tobacco suggested that JAZ proteins regulated the progression of cell death during host and nonhost interactions [23].

Recently, several lines of direct evidence supported that TIFY genes also fulfilled important function in plant responses to environmental challenges [32], [33]. Overexpression of *OsTIFY11a* resulted in increased tolerance to salt and dehydration stresses [15]. Furthermore, OsTIFY3 acted as a transcriptional regulator of the OsbHLH148-mediated JA signaling pathway leading to drought tolerance [34]. Although a series of studies have demonstrated the biological function of TIFYs in salt and drought tolerance, little evidence is given on their roles in alkaline stress responses.

*Glycine soja*, is a wild soybean species and belongs to the same family *Leguminosae* with *Medicago sativa*. *Glycine soja* has extreme excellent tolerance to salt-alkaline stress [35], which makes it as an ideal candidate for exploring resistant genes and breeding of transgenic legume crops with superior salt-alkaline tolerance. In previous studies, we constructed a transcriptional profile of *Glycine soja* (G07256) roots in response to alkaline stress (50 mM NaHCO_3_, pH8.5) [36], and identified three TIFY genes *GsTIFY10a*, *GsTIFY10e* (also named as *GsJAZ2*) and *GsTIFY11b* as alkaline stress responsive genes. We further demonstrated that overexpression of *GsTIFY10a* and *GsTIFY10e* in *Arabidopsis* improved plant alkaline tolerance [37], [38]. In contrast, *GsTIFY11b* overexpression led to decreased salt tolerance [39]. A genomic analysis revealed 34 TIFY genes in *Glycine soja* genome and these GsTIFY proteins were clustered into two groups [40]. Group I comprises 9 members containing a GATA zinc-finger domain (GsTIFY1a, 1b, 1c, 1d, 2a, 2b, 2c, 2d, and 2e), and group II consists of 25 members without GATA zinc-finger domains. Among the group II TIFY proteins, GsTIFY10s (10a, 10b, 10c, 10d, 10e, and 10f) and GsTIFY11s (11a and 11b) were clustered together into one subgroup, here designated as GsTIFY10/11 subgroup. Transcriptional profiles revealed that all GsTIFY10/11 members were dramatically up-regulated at the early stage of alkaline stress, indicating potential roles of GsTIFY10/11s in alkaline stress responses.

In this study, we aimed to identify the regulatory roles and possible physiological and molecular basis of the TIFY10 proteins in plant responses to alkaline stress. We verified the positive function of TIFY10s in alkaline responses by using the *AtTIFY10a* and *AtTIFY10b* knockout Arabidopsis. We also demonstrated the increased alkaline tolerance of transgenic alfalfa ectopically expressing *GsTIFY10a*, and investigated the physiological basis by which *GsTIFY10a* overexpression conferred to increased alkaline tolerance. Finally, we determined the transcriptional activity and dimerization characteristics of GsTIFY10a and GsTIFY10e in yeast and plant cells. Taken together, our results provided direct evidence that TIFY10 proteins positively regulated plant alkaline stress responses.

## Results

### The TIFY10/11 subgroup in Glycine soja and *Arabidopsis thaliana*

**Figure 1:**
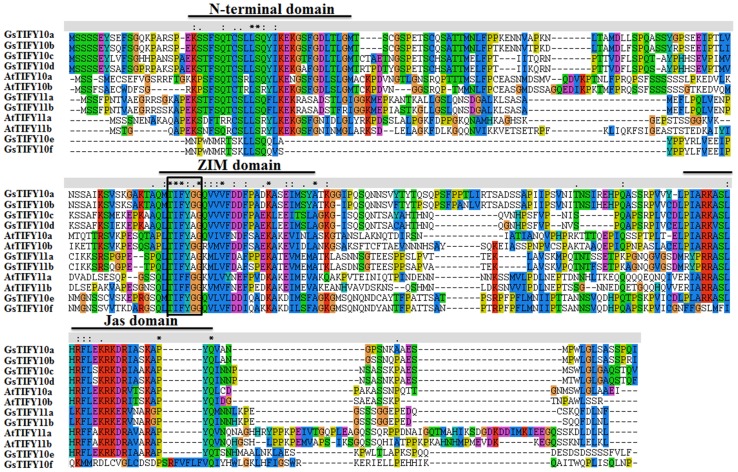
Sequence alignment of the Arabidopsis and wild soybean TIFY10/11 subgroup members based on the full-length amino acid sequences. The conserved N-terminal domain, ZIM domain and Jas domain were marked as solid lines. The TIFY motif (TIFF/YXG) was marked as a black solid box. Sequences were aligned by using ClustalX, and gaps were introduced to maximize alignment.

**Figure 2:**
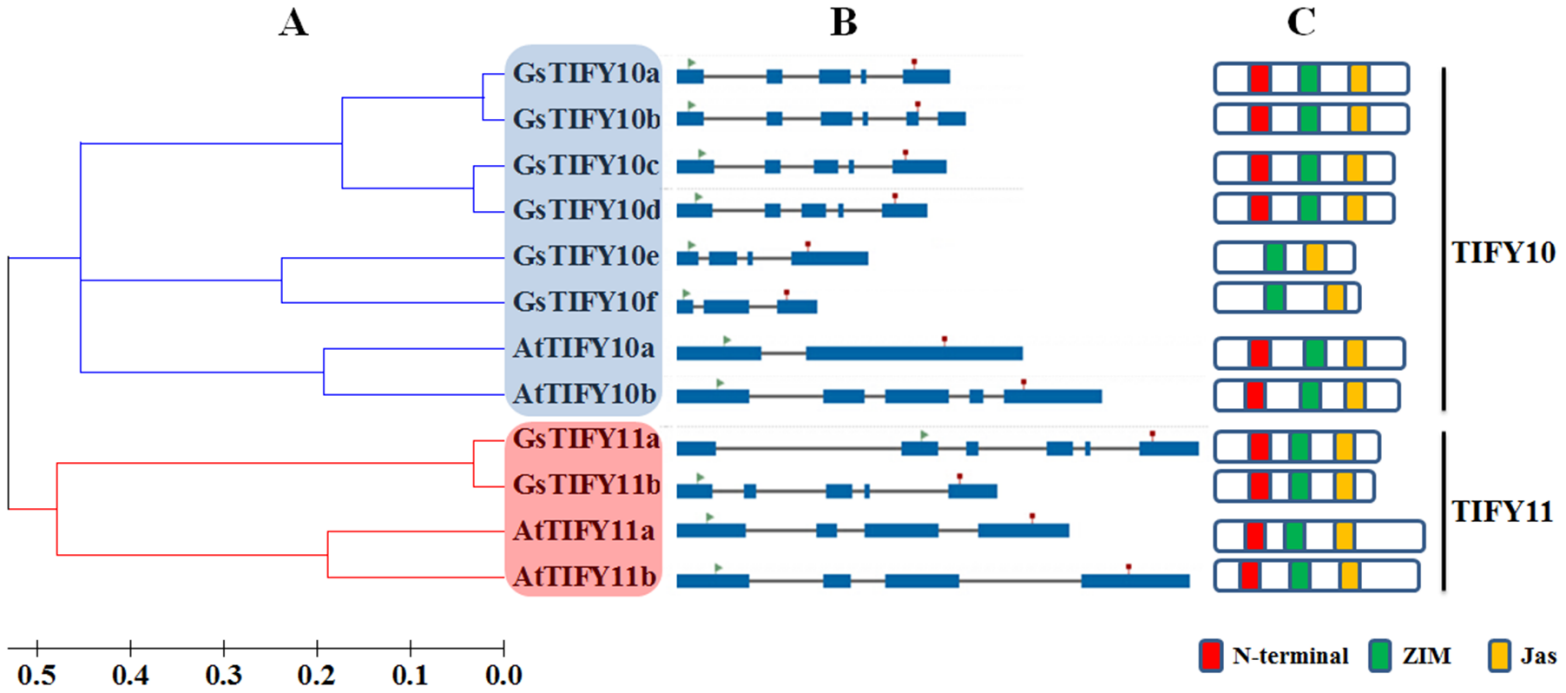
The Arabidopsis and wild soybean TIFY10/11 subgroup proteins. **a**. Phylogenetic analysis of the Arabidopsis and wild soybean TIFY10/11 subgroup proteins. A neighbor-joining tree was constructed with the full-length TIFY10/11 protein sequences by using MEGA 5.0. **b**. Exon/intron structures of the Arabidopsis and wild soybean TIFY10/11 genes. Exons were represented by blue boxes, and grey lines connecting two exons represented introns. Both the exons and introns were drawn to scale. **c**. The distribution of conserved domains within Arabidopsis and wild soybean TIFY10/11 proteins. The relative positions of each conserved domain within each protein were shown in color.

As shown in Fig. 1, the TIFY10/11 subgroup comprises 8 members in *Glycine soja* and 4 members in *Arabidopsis*. Protein sequence analyses showed that all TIFY10/11 proteins contained two highly conserved domains: a ZIM/TIFY domain which mediated homo- and hetero-dimerization, and a Jas domain which played a critical role in repression of JA signaling. It is worth noted that except for GsTIFY10e and GsTIFY10f, all other TIFY10/11 proteins included an N-terminal domain (Fig. 1, Fig. 2C). We further examined the phylogenetic relationship of TIFY10/11s in *Glycine soja* and *Arabidopsis thaliana*. As shown in Fig. 2A, TIFY10/11 proteins were divided into two branches (TIFY10 and TIFY11). GsTIFY10a and GsTIFY10b, GsTIFY10c and GsTIFY10d, GsTIFY10e and GsTIFY10f were closely related with each other, respectively. Among the TIFY10 proteins, GsTIFY10a/b/c/d exhibited high similarity in exon distribution (Fig. 2B), amino acid sequence and domain architecture (Fig. 2C).

To get better understanding of TIFY10/11s sequence diversity, we analyzed the species and numbers of the cis-regulatory elements in their promoter regions. A series of typical elements related to environmental stress, hormone responsiveness and transcription factor (TF) binding sites were identified (Table 1), indicating the involvement of TIFY10/11s in hormone-dependent environmental stress responses and the regulation of TFs on TIFY10/11 expression or activity. Among the 33 elements listed in Table 1, several elements, including ABRELATERD1, ABRERATCAL, ACGTATERD1, ARR1AT, CACGTGMOTIF, DPBFCOREDCDC3, GAREAT, GT1GMSCAM4 and MYCCONSENSUSAT, were found in all of the TIFY10/11s promoter regions. We also noticed several elements only existed in specific TIFY10/11 promoters, for example ABREATRD22, ABREOSRAB21, ABREMOTIFAOSOSEM, ACGTABREMOTIFA2OSEM, ACGTCBOX and ACGTTBOX.

**Table 1:** Sequence diversity and cis-regulatory elements of soybean and Arabidopsis TIFYs promoters.

No.	Site Name	Signal Sequence	Description	GsTIFY10/11s								AtTIFY10/11s			
				10a	10b	10c	10d	10e	10f	11a	11b	10a	10b	11a	11b
**1**	**ABREATRD22**	**RYACGTGGYR**	**ABRE in Arabidopsis dehydration-responsive gene rd22**			**1**	**1**								
**2**	**ABRELATERD1**	**ACGTG**	**ABRE-like sequence required for etiolation-induced expression of erd1 in ** ***Arabidopsis***	**3**	**4**	**2**	**3**	**4**	**5**	**2**	**2**	**5**	**7**	**5**	**6**
**3**	**ABREOSRAB21**	**ACGTSSSC**	**ABRE of wheat Em and rice rab21 genes**		**1**								**1**		
**4**	**ABREMOTIFAOSOSEM**	**TACGTGTC**	**ABRE-like sequence found in rice Osem gene promoter**											**1**	
**5**	**ABRERATCAL**	**MACGYGB**	**ABRE-related sequence in the upstream regions of Ca^2+^-responsive upregulated genes**	**2**	**4**	**2**	**2**	**3**	**3**	**1**	**2**	**7**	**4**	**3**	**5**
**6**	**ACGTABREMOTIFA2OSEM**	**ACGTGKC**	**Sequence requirement of ACGT-core of motif A in ABRE of the rice gene, OSEM**										**1**	**1**	**2**
**7**	**ACGTATERD1**	**ACGT**	**ACGT sequence required for etiolation-induced expression of erd1 in ** ***Arabidopsis***	**4**	**6**	**6**	**4**	**6**	**8**	**4**	**2**	**12**	**18**	**12**	**8**
**8**	**ACGTCBOX**	**GACGTC**	**“C-box” according to the nomenclature of ACGT elements**								**2**				
**9**	**ACGTTBOX**	**AACGTT**	**“T-box” according to the nomenclature of ACGT elements**											**2**	
**10**	**ARFAT**	**TGTCTC**	**ARF binding site found in the promoters of primary/early auxin response genes; AuxRE; enriched in the 5'-flanking region of genes up-regulated by both IAA and BL**	**1**	**1**							**2**	**1**		
**11**	**ARR1AT**	**NGATT**	**“ARR1-binding element” found in Arabidopsis (CK)**	**11**	**4**	**8**	**5**	**6**	**5**	**7**	**11**	**13**	**13**	**10**	**6**
**12**	**ASF1MOTIFCAMV**	**TGACG**	**ASF-1 binding site; are found in many promoters and are involved in transcriptional activation of several genes by auxin and/or salicylic acid**		**1**		**1**		**2**	**1**		**3**	**1**	**1**	**2**
**13**	**BIHD1OS**	**TGTCA**	**Binding site of OsBIHD1, a rice BELL homeodomain transcription factor (disease)**	**4**	**2**	**3**	**1**	**4**	**4**	**1**			**1**	**2**	**5**
**14**	**CACGTGMOTIF**	**CACGTG**	**“CACGTG motif”; “G-box”; Binding site of Arabidopsis GBF4**	**2**	**2**	**2**	**2**	**2**	**2**	**2**	**2**	**2**	**4**	**2**	**4**
**15**	**CACGCAATGMGH3**	**CACGCAAT**	**Sequence found in D4 element in Soybean GH3 gene promoter; Confers auxin inducibility**			**1**									
**16**	**CATATGGMSAUR**	**CATATG**	**Sequence found in soybean SAUR15A promoter; Involved in auxin responses**				**2**					**2**			
**17**	**CBFHV**	**RYCGAC**	**Binding site of barley CBF1 and CBF2**	**1**	**1**			**1**	**1**	**2**		**2**		**1**	**1**
**18**	**CGCGBOXAT**	**VCGCGB**	**“CGCG box” recognized by AtSR1-6 (Arabidopsis thaliana signal-responsive genes)**		**2**							**4**	**2**	**2**	
**19**	**CPBCSPOR**	**TATTAG**	**The sequence critical for Cytokinin-enhanced Protein Binding in vitro**		**1**	**3**	**3**		**2**	**1**	**2**	**2**			**3**
**20**	**CRTDREHVCBF2**	**GTCGAC**	**Preferred sequence for AP2 transcriptional activator HvCBF2; Core CRT/DRE motif**							**2**		**2**			
**21**	**DPBFCOREDCDC3**	**ACACNNG**	**A novel class of bZIP transcription factors binding core sequence (ABA)**	**1**	**1**	**1**	**1**	**1**	**1**	**1**	**2**	**3**	**5**	**2**	**3**
**22**	**DRE2COREZMRAB17**	**ACCGAC**	**“DRE2” core found in maize rab17 gene promoter; rab17 is induced by ABA**	**1**								**1**		**1**	
**23**	**DRECRTCOREAT**	**RCCGAC**	**Core motif of DRE/CRT cis-acting element in many genes in ** ***Arabidopsis*** ** and rice**	**1**	**1**									**1**	**1**
**24**	**ERELEE4**	**AWTTCAAA**	**“ERE (ethylene responsive element)” of tomato E4 and carnation GST1 genes; ERE motifs mediate ethylene-induced activation of the U3 promoter region**	**1**	**1**		**1**					**1**		**2**	
**25**	**GADOWNAT**	**ACGTGTC**	**Sequence present in 24 genes in the GA-down regulated d1 cluster (106 genes) found in Arabidopsis seed germination; This motif is similar to ABRE**										**1**	**1**	**2**
**26**	**GARE1OSREP1**	**TAACAGA**	**“Gibberellin-responsive element (GARE)” found in rice cystein proteinase promoter**			**1**						**1**	**2**		**1**
**27**	**GAREAT**	**TAACAAR**	**GARE (GA-responsive element)**	**1**	**2**	**3**	**1**	**2**	**1**	**1**	**2**	**1**	**3**	**1**	**1**
**28**	**GT1GMSCAM4**	**GAAAAA**	**“GT-1 motif” found in the promoter of soybean CaM isoform, SCaM-4; Plays a role in pathogen- and salt-induced SCaM-4 gene expression**	**5**	**4**	**1**	**3**	**3**	**4**	**3**	**3**	**1**	**2**	**3**	**3**
**29**	**LTRECOREATCOR15**	**CCGAC**	**Core of low temperature responsive element of cor15a gene in Arabidopsis**	**1**	**1**	**1**								**2**	**3**
**30**	**MYB1AT**	**WAACCA**	**MYB recognition site found in the promoters of the dehydration-responsive gene rd22**	**2**	**3**	**1**	**3**	**3**	**2**		**1**		**3**	**2**	**2**
**31**	**MYB2CONSENSUSAT**	**YAACKG**	**MYB recognition site found in the promoters of the dehydration-responsive gene rd22**	**1**				**1**	**1**	**1**			**6**	**2**	**3**
**32**	**MYBCORE**	**CNGTTR**	**Binding site plant MYB proteins ATMYB1 and ATMYB2 from ** ***Arabidopsis***	**1**		**1**	**3**	**4**	**4**	**1**		**1**	**8**	**4**	**6**
**33**	**MYCCONSENSUSAT**	**CANNTG**	**MYC recognition site found in the promoters of the dehydration-responsive gene rd22**	**8**	**8**	**6**	**6**	**10**	**14**	**2**	**4**	**6**	**14**	**4**	**8**

We further checked the responses of GsTIFY10/11s expression to alkaline stress based on the RNA-seq data. Our results revealed that expression of all the GsTIFY10/11 genes was rapidly and greatly induced by alkaline stress. They showed similar alkaline induced expression patterns, with a maximum point at 1 h after NaHCO_3_ treatment (Fig. S1A). Among them, *GsTIFY10e* and *GsTIFY10f* exhibited the greatest alkaline stress induction, while *GsTIFY10a* and *GsTIFY10b* showed the highest expression levels (Fig. S1B). Taken together, these results strongly suggested the potential roles of GsTIFY10/11 proteins in alkaline stress responses.

### The *AtTIFY10a/b* knockout decreased alkaline tolerance at the seed germination stage

**Figure 3:**
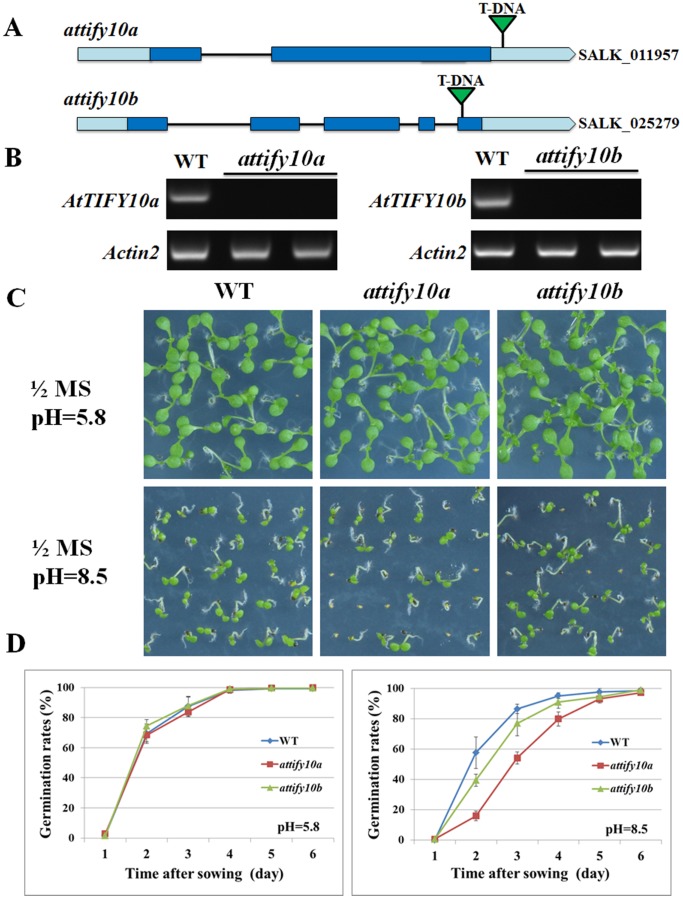
The *AtTIFY10a/b* knockout mutant *Arabidopsis* showed decreased alkaline tolerance at the seed germination stage. **a**. Schematic representation of the *AtTIFY10a/b* T-DNA insertion mutant lines. The exons and introns of the *AtTIFY10a/b* genes were showed as boxes and lines, and the T-DNA insertion sites were marked as triangles. **b**. RT-PCR analyses showing that *AtTIFY10a/b* did not expressed in the *attify10a*/*b* mutants. **c**. The growth performance of WT, *attify10a* and *attify10b* mutant Arabidopsis under alkaline stress. *Arabidopsis* seeds were germinated and grown on 1/2MS medium at pH5.8 or pH8.5. Photographs were taken 6 days after germination. **d**. Seed germination rates of WT and mutant lines. Seeds were considered to be germinated when the radicles completely penetrated the seed coats. A total of 90 seeds from each line were used for each experiment. Data are means (±S.E.) of three replicates.

To confirm the regulatory roles of TIFY10 proteins in alkaline stress responses, we adopted the T-DNA insertion mutant *Arabidopsis* of *AtTIFY10a* (*attify10a*, SALK_011957) and *AtTIFY10b* (*attify10a*, SALK_025279) (Fig. 3A). PCR-based analysis demonstrated the homozygous T-DNA insertion in the *attify10a* and *attify10b* mutants, and RT-PCR results confirmed that the *AtTIFY10a* and *AtTIFY10b* genes did not express in the *attify10a* and *attify10b* mutants, respectively (Fig. 3B).

The wild-type (WT) and mutant Arabidopsis seeds were germinated and grown on 1/2MS solid medium at pH5.8 (Control) or pH8.5 (Alkaline stress), respectively. As shown in Fig. 3C, WT and *AtTIFY10a/b* knockout Arabidopsis seedlings showed similar growth on 1/2MS solid medium at pH5.8, but growth of mutant seedlings was inhibited more severely than that of WT on 1/2MS solid medium at pH8.5. Under alkaline stress, WT seeds maintained relatively high germination rates (86.5%) on the 3^rd^ day, but the germination rates of *attify10a* and *attify10b* seeds dropped to 54.2% and 77.0%, respectively (Fig. 3D). These results suggested that alkaline tolerance of *AtTIFY10a/b* knockout decreased at the seed germination stage, and further confirmed the positive roles of TIFY10 proteins in alkaline stress responses.

### *GsTIFY10a* overexpression in alfalfa enhanced plant alkaline tolerance

**Figure 4:**
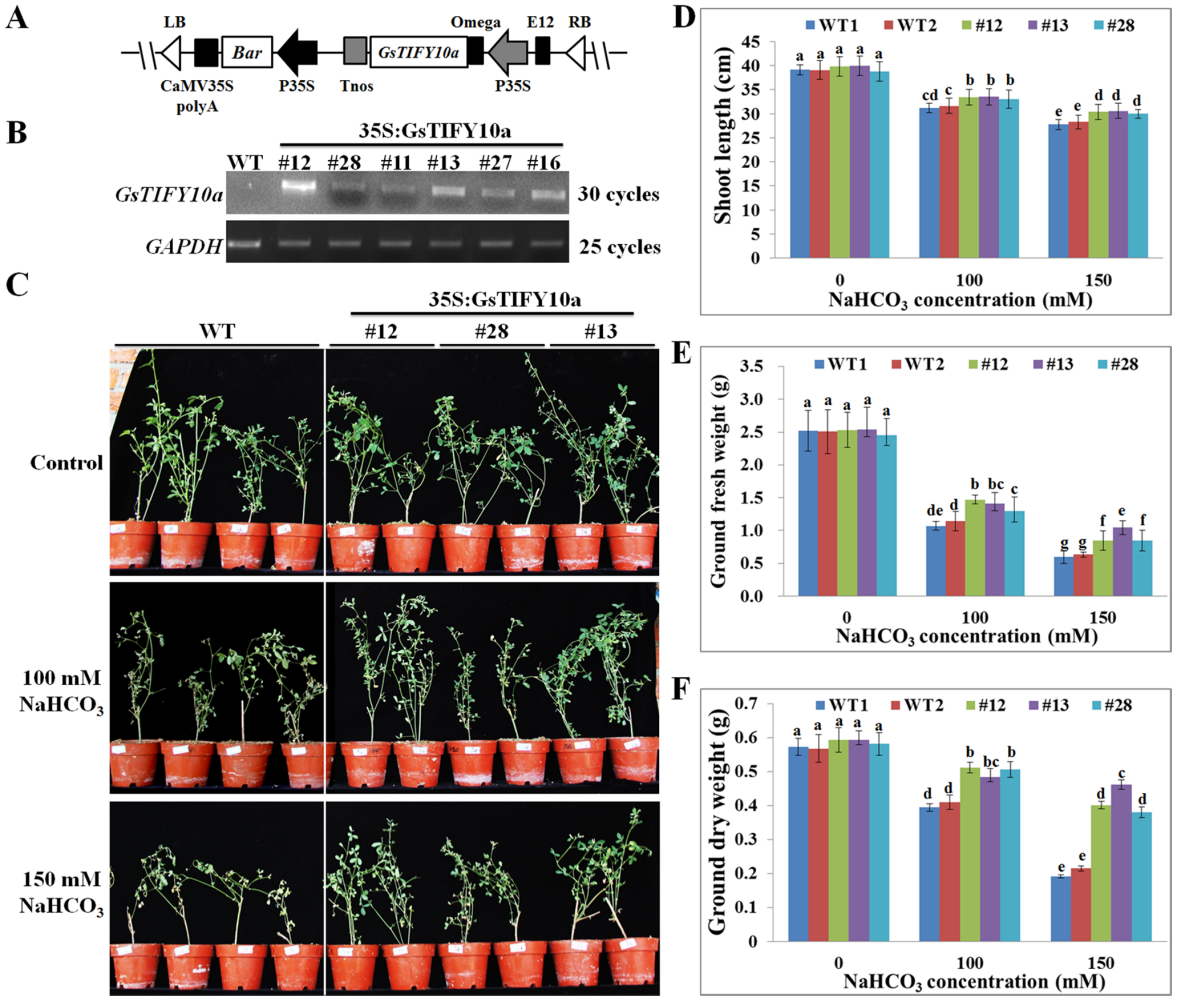
Overexpression of *GsTIFY10a* in alfalfa promoted plant growth under alkaline stress. **a**. Schematic representation of expression constructs to ectopically express *GsTIFY10a* in Medicago *sativa*. **b**. Semi-quantitative RT-PCR analysis showing the transcript levels of *GsTIFY10a* in transgenic alfalfa lines. **c**. Growth performance of WT and transgenic lines under control conditions or NaHCO_3_ treatments. **d**. The shoot length of WT and transgenic plants. **e**. The ground fresh weight of WT and transgenic plants. **f**. The ground dry weight of WT and transgenic plants. For phenotypic analysis under alkaline stress, the propagated WT and *GsTIFY10a* transgenic plants with similar sizes (approximately 25 cm high) were treated with 1/8 Hoagland nutrient solution containing either 0, or 100, or 150 mM NaHCO_3_ every 3 days for a total of 12 days. Photographs were taken 12 days after initial treatment. Thirty plants of each line were used for each experiment. Data are means (±SE) of three replicates. Significant differences were determined by one-way ANOVA (P<0.0001) statistical analysis. Different letters show significant differences between groups as indicated by Dunnett's posttests (P<0.05).

In previous studies, we have demonstrated the involvement of three GsTIFY genes in salt-alkaline stress, among which *GsTIFY10a* could dramatically improve the alkaline tolerance. In an attempt to generate transgenic alfalfa with superior alkaline tolerance, we ectopically expressed *GsTIFY10a* in the wild type *Medicago sativa* through the *Agrobacterium tumefaciens*-mediated transformation strategy. The *GsTIFY10a* gene was under the control of the cauliflower mosaic virus (CaMV) 35S promoter, with the binding enhancers E12 and omega (Fig. 4A). After glufosinate selection, the regenerated alfalfa seedlings were analyzed by PCR and semi-quantitative RT-PCR assays. We identified a total of six transgenic lines (Fig. 4B), and three of them, with different expression levels (#12, #13 and #28), were used to examine the responses to alkaline stress.

We first compared the growth performance of WT and *GsTIFY10a* transgenic alfalfa plants under alkaline stress. As shown in Fig. 4C, under control conditions, transgenic lines showed no obvious differences in seedling growth compared with WT. After 100 or 150 mM NaHCO_3_ treatment for 14 d, both WT and transgenic lines showed growth retardation in a dose-dependent manner. However, the growth inhibition of transgenic lines was less severe than that of WT. After 150 mM NaHCO_3_ treatment, all the transgenic lines could maintain continous growth, whereas WT plants showed severe chlorosis (Fig. 4C). In details, the shoot length, ground fresh weight and dry weight of both WT and transgenic alfalfa plants were decreased gradually with increased NaHCO_3_ concentration, but transgenic lines were much taller (Fig. 4D) and displayed more biomass accumulation (Fig. 4E and 4F) than WT. These results suggested that overexpression of *GsTIFY10a* in alfalfa promoted plant growth under alkaline stress.

**Figure 5:**
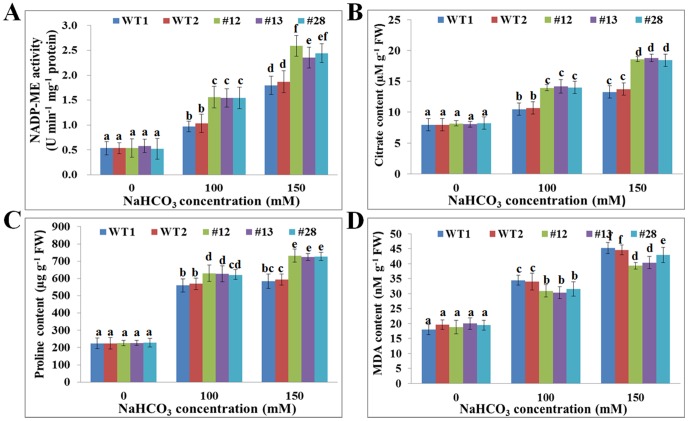
*GsTIFY10a* overexpression altered several physiological indices of transgenic plants under alkaline stress. **a**. The NADP-ME activity of WT and transgenic lines. **b**. The citric acid content of WT and transgenic lines. **c**. The free proline content of WT and transgenic lines. **d**. The MDA content of WT and transgenic lines. Thirty plants of each line were used for each experiment. Data are means (±SE) of three replicates. Significant differences were determined by one-way ANOVA (P<0.0001) statistical analysis. Different letters show significant differences between groups as indicated by Dunnett's posttests (P<0.05).

To further elucidate the influence of *GsTIFY10a* overexpression in alfalfa, several alkaline stress-related physiological and biochemical parameters were analyzed in both WT and transgenic plants, respectively. It is generally accepted that alkaline stress is characterized by high pH value, and always causes much stronger inhibition of plant growth than salt stress [41]. Previous studies showed that NADP-ME could help to maintain the cytosolic pH homeostasis[42], and citric acid was an indicator of plant responses to pH challenge [43]. Therefore, we investigated the NADP-ME activity and citrate acid content of WT and transgenic plants, in an attempt to understand the physiological mechanisms responsible for the increased alkaline tolerance of *GsTIFY10a* transgenic alfalfa. As shown in Fig. 5A and 5B, alkaline stress obviously improved the NADP-ME activity and citrate acid content in both WT and transgenic lines, however, an obvious up-regulation was observed in the transgenic lines. These results indicated that the alleviation of high pH damage in *GsTIFY10a* transgenic alfalfa might partially result from the ability to maintain the cytosolic pH homeostasis through increased NADP-ME activity and citrate acid content.

As a type of compatible osmolyte, proline plays a critical role in protecting plants from environmental stresses [44]. Our results revealed that transgenic alfalfa plants accumulated more free proline than WT in the presence of 100 or 150 mM NaHCO_3_ (Fig. 5C). To further test the cell membrane stability, we further determined the malon dialdehyde (MDA) content of WT and transgenic plants. Under control conditions, the MDA content of transgenic lines was similar to that of WT (Fig. 5D). After NaHCO_3_ treatment for 14 d, the MDA content of WT were significantly higher than that of transgenic alfalfa. Collectively, these results demonstrated that the increased alkaline tolerance of *GsTIFY10a* transgenic alfalfa might be related to the elevated levels of NADP-ME activity, citrate content and proline content, as well as reduced MDA content.

### *GsTIFY10a* overexpression up-regulated the expression levels of stress responsive genes

**Figure 6:**
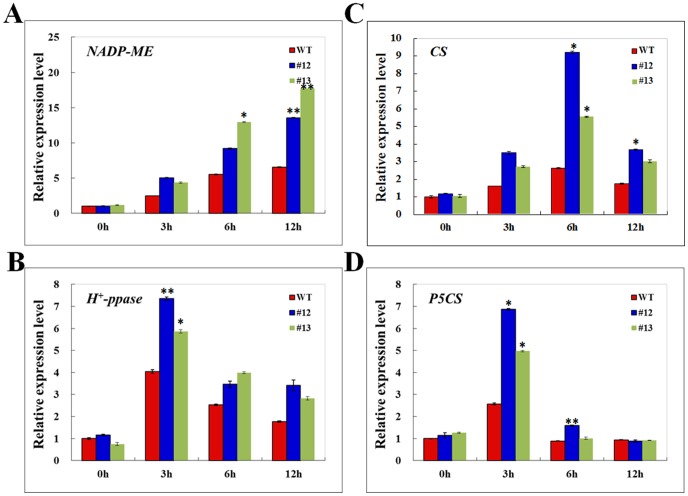
*GsTIFY10a* overexpression up-regulated the expression levels of several stress responsive genes. **a**. Increased expression levels of *NADP-ME* in transgenic plants under alkaline stress. **b**. Increased expression levels of *CS* in transgenic plants. **c**. Increased expression levels of *H^+^-ppase* in transgenic plants. **d**. Increased expression levels of *P5CS* in transgenic plants. To explore the expression patterns of stress-responsive genes, the 4-week-old WT and transgenic seedlings (line #12 and #13) after shoot cottage were treated with 1/8 Hoagland solution containing 50 mM NaHCO_3_ (pH 8.5) for 0, 3, 6, and 12 h, respectively. Relative transcript levels were determined by quantitative real-time PCR with the *MtGAPDH* gene as an internal reference, and were normalized to WT at 0 h. Values represented the means of three independent biological replicates, and three technological replicates for each. *P<0.05; **P<0.01 by Student's t-test.

As described above, *GsTIFY10a* overexpression increased the NADP-ME activity, citrate content and proline content of transgenic alfalfa. To explore the molecular basis of *GsTIFY10a* in the cytoplasmic pH regulation and osmotic regulation under alkaline stress, we examined the expression levels of *NADP-ME*, *CS*, *H^+^-Ppase*, and *P5CS* in WT and two transgenic lines (#12 and #13). The real-time PCR results showed that their expression was greatly induced by alkaline stress in both WT and transgenic lines (Fig. 6). Expectedly, their expression levels in transgenic plants were significantly higher than that in WT, which explained the up-regulation of the NADP-ME activity, citrate acid content and free proline content in transgenic lines. These results also implied that *GsTIFY10a* overexpression promoted the transcript accumulation levels of the stress responsive genes, which might be helpful for the intracellular pH homeostasis and osmotic regulation under alkaline stress.

### Increased JA content in *GsTIFY10a* transgenic lines

**Figure 7:**
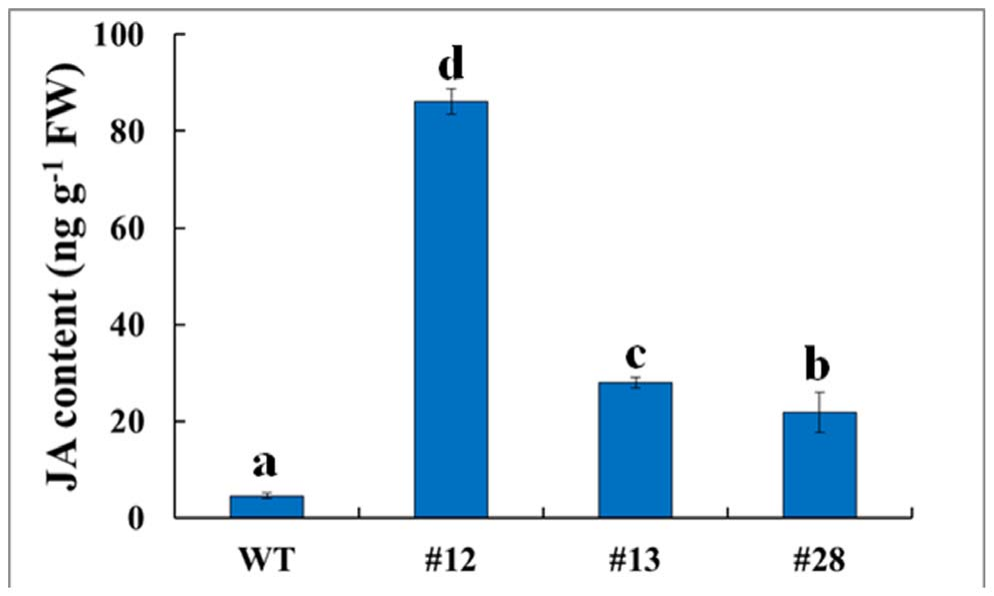
Increased JA content in *GsTIFY10a* transgenic plants. Leaves of WT and transgenic plants were harvested for JA extraction, and subjected for HPLC analysis to determine the content of endogenous JA. Each data point represents the mean (±SE) of three samples from independent sets of plants. Significant differences were found using one-way ANOVA analysis (P = 1.07*10^−9^).

TIFY/JAZ proteins are repressors of JA signaling in plants. Our previous studies revealed that *GsTIFY10a* overexpression in Arabidopsis repressed the transcription of JA responsive genes [38]. Hence, in this study, we determined the JA content in the transgenic alfalfa lines. As shown in Fig.7, JA contents were significantly increased in the transgenic alfalfa lines (F_3,8_ = 581.042, P = 1.07*10^−9^).

### Transcriptional activity assays of GsTIFY10a and GsTIFY10e

**Figure 8:**
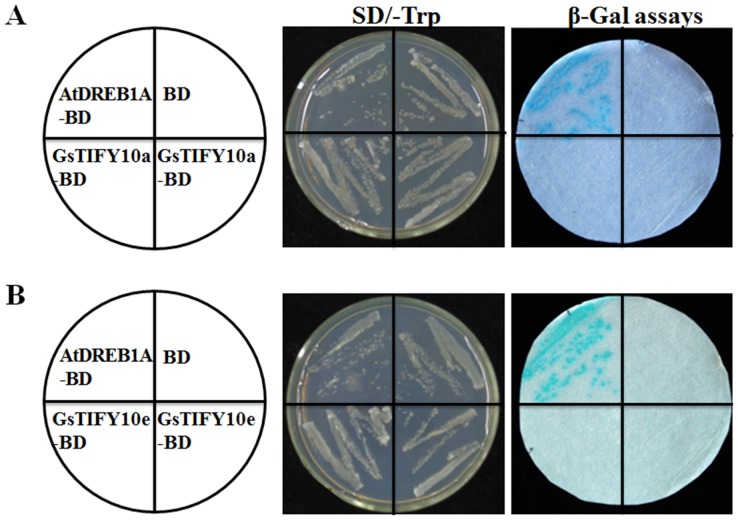
Transcriptional activity analysis of the GsTIFY10a and GsTIFY10e proteins. **a**. Transcriptional activity analysis of GsTIFY10a. **b**. Transcriptional activity analysis of GsTIFY10e. The pGBKT7-GsTIFY10a and pGBKT7-GsTIFY10e vectors were transformed into the yeast reporter strain AH109, and LacZ activity was assessed by using the β-galactosidase filter lift assays. The AtDREB1A transcription factor was used as a positive control.

Our previous studies demonstrated that two of the *Glycine soja* TIFY10 proteins GsTIFY10a and GsTIFY10e positively regulated plant tolerance to alkaline stress. Both of them were found to localize at the nuclei of plant cells, indicating that they might act as transcriptional regulators. To further investigate the molecular basis of the TIFY10 proteins in alkaline responses, we identified the transcriptional activity of the GsTIFY10a and GsTIFY10e proteins. The full-length *GsTIFY10a* and *GsTIFY10e* genes were fused to the GAL4 DNA-binding domain in the pGBKT7 vector, and then introduced to the yeast reporter strain AH109. The AtbZIP1 transcription factor, which showed transcriptional activity in yeast cells, was used as a positive control [45], [46]. LacZ activity was assessed by using the β-galactosidase filter lift assays. As shown in Fig.8, only the recombinant yeast cells carrying the AtbZIP1-BD vector displayed LacZ activity. These results implied that neither GsTIFY10a nor GsTIFY10e showed transcriptional activity in yeast cells.

### Dimerization analyses between GsTIFY10a and GsTIFY10e

**Figure 9:**
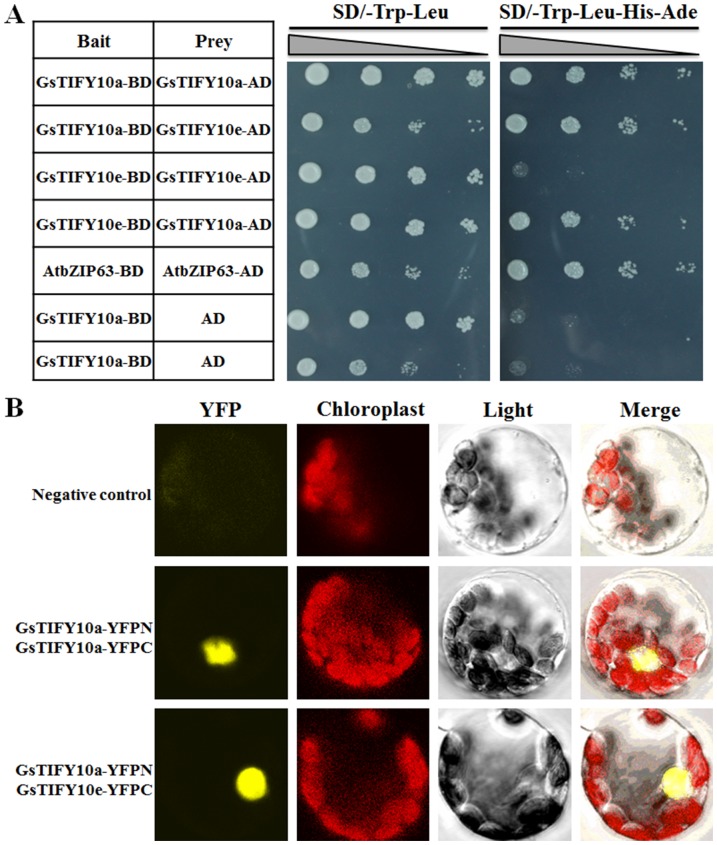
Dimerization assays of the GsTIFY10a and GsTIFY10e proteins. **a**. Dimerization analyses between GsTIFY10a and GsTIFY10e in yeast cells. Pictures showed the growth performance of recombinant yeast cells harboring different plasmids on SD/-Trp-Leu and SD/-Trp-Leu-Ade-His medium. The AtbZIP63-BD/AtbZIP63-AD combination was used as a positive control, and the GsTIFY10a-BD/AD and GsTIFY10e-BD/AD combinations were used as negative controls. **b**. Dimerization analyses between GsTIFY10a and GsTIFY10e in living plant cells. The YFPN/YFPC combination was used as a negative control. Pictures showed the YFP fluorescence, chlorophyll auto-fluorescence, light and overlay visions.

It has been suggested that the ZIM domains mediated homo- and heterodimerization of the TIFY proteins [47]. To verify if GsTIFY10a and GsTIFY10e could form homodimers or heterodimers with each other, we performed the yeast two hybrid analyses. The AtbZIP63 transcription factor, which formed homodimers in plant cells [48], was used as a positive control. As shown in Fig.9A, the yeast cells carrying GsTIFY10a-BD/GsTIFY10a-AD, GsTIFY10a-BD/GsTIFY10e-AD, GsTIFY10e-BD/GsTIFY10a-AD and AtbZIP63-BD/AtbZIP63-AD (positive control) were capable of growth on the both SD/-Trp-Leu and SD/-Trp-Leu-Ade-His medium. However, the yeast cells harboring GsTIFY10e-BD/GsTIFY10e-AD, GsTIFY10a-BD/AD (negative control), GsTIFY10e-BD/AD (negative control) could not grow on the SD/-Trp-Leu-Ade-His medium. These results demonstrated that GsTIFY10a, not GsTIFY10e, could form homodimers, and GsTIFY10a and GsTIFY10e could heterodimerize with each other in yeast cells.

To further verify their physical interaction in living plant cells, we performed the bimolecular fluorescence complementation (BiFC) assays. To this end, we fused GsTIFY10a to the N-terminal YFP fragment and GsTIFY10a/e to the C-terminal YFP fragment, to generate GsTIFY10a-YFPN and GsTIFY10a/e-YFPC constructs, respectively. GsTIFY10a-YFPN/GsTIFY10a-YFPC and GsTIFY10a-YFPN/GsTIFY10e-YFPC were co-transformed into *Arabidopsis* protoplasts, respectively, and the empty YFPN/YFPC was used as a negative control. As shown in Fig. 9B, YFP fluorescence was observed from protoplasts co-transformed with GsTIFY10a-YFPN/GsTIFY10a-YFPC and GsTIFY10a-YFPN/GsTIFY10e-YFPC, but not from YFPN/YFPC. The BiFc results confirmed the homodimerization of GsTIFY10a, as well as the heterodimerization between GsTIFY10a and GsTIFY10e in plant cells.

## Discussions

The TIFY protein family, characterized by a highly conserved TIFY motif, constitutes a particular class of plant-specific transcription factors with a broad range of biological functions. According to their distinct domain architectures, TIFY proteins could be classified into four subfamilies: the TIFY subfamily containing only the TIFY domain, the JAZ subfamily containing the TIFY domain and the Jas domain, the PPD subfamily containing the PPD, TIFY and a truncated Jas domain, and the ZML subfamily containing the TIFY, CCT and ZML domains[14]. Among them, the JAZ subfamily proteins could be further clustered into five groups (group I-V) [14]. The first group (group I) is composed of the TIFY10 and TIFY11 proteins. In this study, we focused on the group I TIFY proteins in Arabidopsis and wild soybean. All of the group I TIFY proteins contained the conserved TIFY and Jas domains (Fig. 1, 2), which was the canonical characteristic of JAZ subfamily. It is noteworthy that except for GsTIFY10e and GsTIFY10f, all other TIFY10/11s included an N-terminal domain (Fig. 1, 2).

The best-characterized TIFY10 protein is the Arabidopsis TIFY10a/JAZ1, which was found to play a critical role in JA signaling. In the absence of JAs, JAZ1 interacts with and represses the downstream transcriptional activators, such as MYC2 [26], MYC3, MYC4 [27], MYB21, MYB24 [28], bHLH017 and bHLH003 [29], which control the expression of JA-responsive genes. In the presence of JAs, AtTIFY10a protein is recognized and degraded by the SCF (COI1) E3 ubiquitin ligase, releasing the downstream transcription factors [49]. Jasmonoyl isoleucine (JA-Ile) and coronatine could promote the physical interaction between JAZ1 and COI1, and the C-terminal Jas domain of JAZ1 is critical for JA-Ile/coronatine-dependent interaction with COI1 [49], [50]. Recently, a transacting factor AtBBD1 was suggested to interact with AtTIFY10a and bind to the JARE element upstream of the JA responsive gene AtJMT [51]. In addition to the negative regulatory role in JA signaling pathway, AtTIFY10a was also reported to be involved in phytochrome A [52] and auxin signaling [53]. Furthermore, AtTIFY10a physically interacted with ICE1 and ICE2 transcription factors, repressed the transcriptional function of ICE1, and thereby affected plant freezing stress responses [33]. However, no direct evidence supporting its role in alkaline stress responses was reported until now.

Recently, the systemic transcriptional analyses and several lines of genetic evidence suggested the potential regulatory roles of TIFY10/11s in plant responses to environmental challenges [15], [33]. Our previous research also revealed the alkaline stress induced expression of *Glycine soja* TIFY10/11 genes [36], [40], which was further confirmed by the RNA-seq data (Fig. S1). Consistently, we also observed some cis-regulatory elements related to environmental stress response in the TIFY10/11s promoters (Table 1). In addition, we also found elements involved in hormone responsiveness, suggesting the crosstalk between environmental stress and hormone signal. Several elements were common to all TIFY10/11s, implying that TIFY10/11 proteins were involved in the same signal transduction pathway. Remarkably, several elements only existed in specific TIFY10/11 promoters, which might indicate the specific response of TIFY10/11s to environmental stress.

Among the eight GsTIFY10/11 genes, overexpression of *GsTIFY10a* and *GsTIFY10e* in *Arabidopsis* obviously improved plant alkaline tolerance [37], [38], while *GsTIFY11b* overexpression led to decreased salt tolerance [39]. In this study, we suggested the positive roles of AtTIFY10a/b in plant tolerance to alkaline stress by using the plate germination assays (Fig. 3), further supporting the important roles of TIFY10s in plant alkaline stress responses. It is worth to note that, under the same alkaline stress treatment, *attify10a* (54.2% on the 3^rd^ day) exhibited relatively lower germination rates than *attify10b* (77.0%), indicating a more important role of *AtTIFY10a* than *AtTIFY10b* in alkaline stress responses.

In the current study, we also transformed the *GsTIFY10a* gene into *Medicago sativa* in an attempt to obtain transgenic alfalfa with superior alkaline tolerance. Our results demonstrated that *GsTIFY10a* overexpression dramatically promoted growth of transgenic plants under alkaline stress. *GsTIFY10a* transgenic lines displayed much better at shoot height, ground fresh weight and dry weight than WT under alkaline stress (Fig. 4), which was in line with the better growth performance of *GsTIFY10a* transgenic Arabidopsis [38]. Furthermore, we also investigated the potential physiological and molecular basis of *GsTIFY10a* in response to alkaline stress (Fig. 5, 6). Firstly, *GsTIFY10a* overexpression could help plant to deal with the high pH damage by up-regulating the NADP-ME activity and citrate acid content (Fig. 5). The up-regulation of *NADP-ME* and *CS* gene expression in transgenic plants might be helpful to explain the increase of NADP-ME activity and citrate acid content (Fig. 6). Meanwhile, we also observed an obvious increase of *H^+^-ppase* expression in transgenic lines under alkaline stress (Fig. 6), which was also helpful for maintaining the cytosolic pH homeostasis. These results were consistent with our previous observation that *GsTIFY10a* overexpression up-regulated the expression of *NADP-ME* and *H^+^-ppase* in Arabidopsis [38]. Secondly, *GsTIFY10a* overexpression led to greater proline accumulation (Fig. 5) and up-regulated expression of the *P5CS* gene (Fig. 6), which encodes a key enzyme in the proline biosynthesis process. Proline serves as a compatible osmolyte, molecular chaperone and ROS scavenger in plant responses to environmental stress [54]. The increased accumulation of free proline in *GsTIFY10a* transgenic lines might be of great importance for the effective osmo-regulation and ROS scavenging of plant cells under alkaline stress. In addition, *GsTIFY10a* transgenic lines displayed lower levels of MDA content under alkaline stress (Fig. 5D). MDA is widely recognized as an indicator for lipid peroxidation resulted from the elevated ROS accumulation in plant cells [55]. The decreased MDA accumulation also indicated the more effective adaptation of transgenic plants to ROS damage caused by alkaline stress. Taken together, we speculated that *GsTIFY10a* overexpression is of fundamental importance for the cytosolic pH regulation, osmo-regulation and ROS scavenging by regulating the related gene expression and enzyme activity, and thereby promoted plant growth under alkaline stress.

A great number of studies have demonstrated that JA signaling is critical for plant stress responses and TIFY proteins are involved in plant tolerance to both biotic and abiotic stresses such as wounding [52], [56], pathogen [23], [57], drought stress [15], [34], and salt stress [20], [32]. Our studies suggested the involvement of TIFYs in alkaline stress responses and the crosstalk between alkaline stress and JA signaling [37], [38], [40]. All of the *GsTIFY10/11* genes were dramatically up-regulated at the early stage of alkaline stress treatment (Fig. S1). Overexpression of GsTIFY10e/JAZ2 significantly improved plant alkaline stress tolerance [37]. Knockout of AtTIFY10a and AtTIFY10b also inhibited seed germination under alkaline stress (Fig. 3). Furthermore, *GsTIFY10a* expression was greatly induced by both alkaline stress and MeJA treatment [38].

In addition to the increased alkaline tolerance, *GsTIFY10a* overexpression in Arabidopsis conferred MeJA insensitivity. *GsTIFY10a* repressed transcription of the JA responsive genes such as *PDF1.2* (Plant Defensin 1.2), *VSP2* (Vegetative Storage Protein 2), *AOS* (Allelen Oxide Synthase) and *LOX2* (Lipoxygenase 2), whose expression was also induced by alkaline stress. However, in the present study, we found that JA contents were significantly increased in the transgenic alfalfa lines (Fig.7). As we know, environmental stress could stimulate the biosynthesis of JAs. The elevated JAs levels promoted the interaction of TIFY/JAZ with SCF^CoI1^, which led to degradation of TIFY/JAZ proteins, and subsequently release of the targeted TFs. Activation of TFs induced transcription of JA-responsive genes, and the newly synthesized TIFY/JAZ proteins could restore the repression of TFs. The *GsTIFY10a* transgenic lines always kept high levels of GsTIFY10a proteins, which repressed the TFs activity and JA signaling. Hence, plants need to synthesize more JAs to degrade the GsTIFY10a proteins. Collectively, these results strongly suggested the crosstalk between alkaline stress and JA signaling. Molecular basis of the hypothesis that TIFY10 proteins mediate alkaline stress responses through JA signaling need be further studied.

The JAZ proteins were suggested to act as transcriptional repressors in JA signaling because they do not contain a known DNA binding domain. Due to the nuclear localization of several JAZ subfamily members [25], [58], it is proposed that they might exert their effect on gene expression through protein interaction with transcription factors. Our previous revealed that, like other JAZs, GsTIFY10a and GsTIFY10e localized in the nuclei of plant cells. In this study, we found that GsTIFY10a and GsTIFY10e showed no transcriptional activity in yeast cells (Fig.8), indicating them as transcriptional repressors. On the other hand, it has been well demonstrated that the ZIM domains of JAZ/TIFYs mediate formation of homo- and heteromeric complexes [59]. For example, the Arabidopsis JAZ1/TIFY10a and JAZ2/TIFY10b could form homodimers and heterodimers [59]. In this study, by using the Y2H and BiFc technologies, we found that GsTIFY10a could form homodimers, as well as heterodimers with GsTIFY10e, while GsTIFY10e could not homodimerize (Fig.9). Similarly, in case of Arabidopsis JAZs, JAZ7, JAZ8, JAZ9, JAZ11, and JAZ12 could not homodimerize, but JAZ9 and JAZ12 could form heterodimers with other JAZs [59].

In summary, here we provide novel insights into the regulatory roles and potential molecular basis of TIFY10 proteins in plant responses to alkaline stress. We obtained the transgenic alfalfa with superior alkaline tolerance by ectopically expressing *GsTIFY10a*, and investigated the possible physiological and molecular mechanism by which *GsTIFY10a* regulated plant alkaline tolerance. For now, the *GsTIFY10a* transgenic alfalfa is at the stage of the “biosafety evaluation of genetically modified organisms”, and will provide a bearing on future aims at salt-alkaline soil management.

## Materials and Methods

### Plant materials and growth conditions

Seeds of the wild type *Arabidopsis thaliana* (Columbia ecotype), *attify10a* (SALK_011957) and *attif10b* (SALK_025279) mutant lines were kindly provided by the European Arabidopsis Stock Centre (UK). Both WT and mutant Arabidopsis seeds were germinated and grown in standard nutrient solution as described by Tocquin et al. (2003). The Arabidopsis seedlings were maintained in a greenhouse under controlled environmental conditions (21–23°C, 100 µmol m^−2^ s^−1^, 60% relative humidity, 16 h light/8 h dark cycles).

Alfalfa (*Medicago sativa* L. cv. Nongjing No. 1, a local species) was used in this study, and was kindly obtained from Heilongjiang Academy of Agricultural Sciences (Harbin, China). Alfalfa was grown in a greenhouse under controlled environmental conditions (24–26°C, 600 µmol m^−2^ s^−1^, 80% relative humidity, 16 h light/8 h dark cycles).

### Seed germination assays of *AtTIFY10a/b* knockout Arabidopsis

Homozygous T-DNA insertion mutant Arabidopsis was obtained by using a PCR-based method as described previously [60]. Briefly, the gene specific primers across the T-DNA insertion sites of *attify10a* and *attif10b* mutant (5′-TGCGATCCAGCCAAAGCGTCT-3′ and 5′-GAGTATTTGATAGTATGGTTCGTCAACAA-3′ for *attify10a*, 5′-TGAGAAGAGGAAGGATAGGTAATGCAT-3′ and 5′-CAAGCTAATGTTGAGATCGGCAGAG-3′ for *attify10b*) were used for homozygous identification. T-DNA insertion was confirmed by PCR amplification with the following primers (5′-TGCGATCCAGCCAAAGCGTCT-3′ and 5′-GATTTGGGTGATGGTTCACGTAGTG-3′ for *attify10a*, 5′-GAGAAGAGGAAGGATAGGTAATGCAT-3′ and 5′-GATTTGGGTGATGGTTCACGTAGTG-3′ for *attify10b*).

RT-PCR analysis was used to confirm the silence of *AtTIFY10a* and *AtTIFY10b* genes in the T-DNA insertion mutants. The following gene specific primers were used (5′-TGCGATCCAGCCAAAGCGTCT-3′ and 5′-GAGTATTTGATAGTATGGTTCGTCAACAA-3′ for *AtTIFY10a*, 5′-TGAGAAGAGGAAGGATAGGATCACATC-3′ and 5′-CAAGCTAATGTTGAGATCGGCAGAG-3′ for *AtTIFY10b*).

For plate germination assays under alkaline stress, seeds of WT and mutant lines were surfaced-sterilized with 5% sodium hypochlorite (NaClO) for 6–8 min with shaking, washed with sterilized distilled water for 6–8 times, and kept at 4°C for 3 days to break seed dormancy. After that, WT and mutant *Arabidopsis* seeds were sown on 1/2 MS agar medium with 1% (w/v) sucrose and 0.8% (w/v) agar at pH5.8 (Control conditions) or pH8.5 (Alkaline stress). The germination percent was recorded for consecutive 6 days after sowing, and pictures were taken to show the growth performance of each line. Ninety seeds from each line were used for each experiment and the experiments were repeated for three times.

### Generation of *GsTIFY10a* overexpression transgenic alfalfa

In order to investigate the effect of *GsTIFY10a* on alkaline stress tolerance, *GsTIFY10a* was cloned into the pBEOM plant expression vector, and was under the control of the cauliflower mosaic virus (CaMV) 35S promoter, with the binding enhancers E12 and omega. The *Bar* gene was used as the selectable marker. The recombinant vector pBEOM-GsTIFY10a was introduced into *Agrobacterium tumefaciens* strain EHA105, and then introduced into *Medicago sativa* by using the cotyledonary node method as described previously [13]. Briefly, alfalfa seeds were surface sterilized with 70% ethanol for 1 min, 0.1% (v/v) HgCl_2_ for 15 min, and then washed with sterilized water for 3–5 times. Sterilized seeds were germinated and grown on 1/2MS medium for 8 days, and then seedlings were aseptically excised at the cotyledonary node position as explants. The explants were infected with *A. tumefaciens* EHA105 for 15 min, and placed vertically into MS medium (pH 5.2) containing 1 mg L^−1^ 6-benzylaminopurine (6-BA) and 100 mM L^−1^ acetosyringone for 3 days. After that, explants were washed in liquid MS medium (pH 5.8) containing 1 mg L^−1^ 6-BA, 0.5 mg L^−1^ glufosinate and 100 mg L^−1^ amoxicillin. Then explants were transferred onto solid MS medium (pH 5.8) containing 1 mg L^−1^ 6-BA, 0.5 mg L^−1^ glufosinate and 100 mg L^−1^ amoxicillin, and grown for another 14 days to form the glufosinate-resistant regenerated shoots. The regnerated shoots were then transferred to new solid medium as described above, and grown for another 14 days. Elongated shoots were then transferred to new 1/2MS medium only containing 100 mg L^−1^ amoxicillin, until roots appeared.

The regenerated alfalfa plants were then transplanted into soil under controlled conditions and confirmed by PCR analysis using CaMV35S promoter specific forward primer and *Bar* gene specific reverse primer (5′-TGCACCATCGTCAACCACTACATCG-3′ and 5′-CCAGCTGCCAGAAACCCACGTCATG-3′). To eliminate the potential existence of the chimeric transgenic plants, new-born leaves from different branches of each regenerated plants were used for PCR identification. The transcript levels of *GsTIFY10a* in the PCR-positive plants were further analyzed by semi-quantitative RT-PCR analyses with gene specific primers (5′-ACAGAGCCAGCCTTCATTTCC-3′ and 5′-CGAACCCGACTCACGAAGAAG-3′). The alfalfa glyceraldehyde-3-phosphate dehydrogenase gene (*MtGAPDH*, Accession: Medtr3g085850) was used as an internal control, and PCR amplified with the following primer pair (5′-GTGGTGCCAAGAAGGTTGTTAT-3′ and 5′-CTGGGAATGATGTTGAAGGAAG-3′).

### Phenotypic analyses of transgenic alfalfa under alkaline stress

For phenotypic analyses under alkaline stress, the lignified WT and transgenic alfalfa plants were used for vegetative propagation through stem cuttings. The propagated seedlings were transplanted into plastic culture pots filled with a mixture of peat moss: soil (1∶1; v/v), irrigated with 1/8 Hoagland nutrient solution and grown in a greenhouse under controlled conditions. To eliminate the potential existence of the chimeric transgenic plants, each propagated seedlings were subjected for PCR identification before stress treatment. PCR-positive plants with similar sizes (approximately 25 cm high) were then exposed to alkaline stress by irrigating with 1/8 Hoagland solution containing either 0, or 100, or 150 mM NaHCO_3_ every 3 days for a total of 12 days. Photographs were taken on the 12^th^ day. Thirty plants of each line were used for each experiment, and the experiments were repeated for at least three times.

The MDA content was determined according to the protocol described by Peever et al. [61]. The citric acid content was determined by using a spectrophotometer (UV-2550, Shimadzu, Japan) at the absorbance of 490 nm according to the method of Zhu [62]. Free proline content was measured according to the method of Bates et al. [63]. NADP-ME activity was measured as described by Geer et al. [64].

All of the above numerical data were subjected to statistical analyses using EXCEL 2010 and/or IBM SPSS statistics 19, and analyzed by Student's T-test and/or one-way ANOVA analysis.

### Quantitative real-time PCR analyses

To investigate the expression patterns of stress-responsive genes, the 4-week-old seedlings after shoot cottage were treated with 1/8 Hoagland solution containing 50 mM NaHCO_3_ (pH 8.5) for 0, 3, 6, and 12 h, respectively. Equal amounts of leaves were harvested and stored at −80°C after snap-frozen in liquid nitrogen.

Total RNA was extracted by using RNeasy Plant Mini Kit (Qiagen, Valencia, CA, USA), and subjected to cDNA synthesis by using SuperScript™ III Reverse Transcriptase kit (Invitrogen, Carlsbad, CA, USA). Quantitative real-time PCR was performed using a Stratagene MX3000P real-time PCR instrument and the SYBR Select Master Mix (Applied Biosystems, USA). The MtGAPDH gene was used as an internal reference. Expression levels of all candidate genes were calculated by using the 2^−ΔΔCT^ method, and the relative intensities were calculated and normalized as described previously 65. Three independent biological replicates were carried out and subjected to real-time PCR in triplicate. Primers used for quantitative real-time PCR were designed by using Primer 5 software and listed in Table 2.

**Table 2:** Gene-specific primers used for quantitative real-time PCR assays.

Gene name	Gene ID	Primer Sequence (5′ to 3′)
*GsGAPDH*	DQ355800	Forward: GACTGGTATGGCATTCCGTGT Reverse: GCCCTCTGATTCCTCCTTGA
*GsTIFY10a*		Forward: TCCAGCGCAATTAAGTCTGTGAG Reverse: TTTGGTGGCATAGGACATGATCT
*MtGAPDH*	Medtr3g085850	Forward: GTGGTGCCAAGAAGGTTGTTAT Reverse: CTGGGAATGATGTTGAAGGAAG
*P5CS*	EU371644.1	Forward: TCGGGGTCCAGTAGGAGTTG Reverse: AGTAGTTAGGTCTTTGTGGGTGTAGG
*H^+^-ppase*	XM_003609415.1	Forward: TCTCCACCGACGCATCTATCA Reverse: GGCATTATCCCAAGCACCG
*NADP-ME*	XM_003630679.1	Forward: TGGCGGTGTTGAGGATGTCT Reverse: CTGACAGTGGGAGGTAAGAGGC
*Citrate Synthase*	HM030734.1	Forward: TTAAAGCCAGGAAACGAAAGC Reverse: AAGGCAACAGCAACCTCAATC

### Transcriptional activation assays

The full-length coding regions of *GsTIFY10a* and *GsTIFY10e* were amplified by using the following primer pairs: 5′-CTTGAATTCTCGAGCTCATCGG-3′ and 5′-GAAGTCGACGATTTGAGGTGAA-3′ (for GsTIFY10a), 5′-CGGGAATTCAATCCATGGAAC-3′ and 5′-GCCGTCGACTAACACAAAGCTGG-3′ (for GsTIFY10e). The PCR products were cloned to the pGBKT7 vector to express the GsTIFY10a-BD and GsTIFY10e-BD fused proteins.

For the transcriptional activation assays, the corresponding vectors pGBKT7 (negative control), pGBKT7-GsTIFY10a, pGBKT7-GsTIFY10e and pGBKT7-AtbZIP1 (positive control) were transformed into yeast strain AH109. The transformants were selected on SD/-Trp medium. LacZ activity was analyzed by using the β-galactosidase filter lift assay.

### Dimerization analyses of GsTIFY10a and GsTIFY10b in yeast cells

The full-length coding regions of *GsTIFY10a* and *GsTIFY10e* were amplified by using the following primer pairs: 5′-GCTCATATGTCGAGCTCATCGG-3′ and 5′-CTTGAATTCGATTTGAGGTGAA-3′ (for *GsTIFY10a*), 5′- GCCCATATGACCTCAGTGGAG-3′ and 5′-GTTGGATCCTAACACAAAGCTGG-3′ (for *GsTIFY10e*). The PCR products were cloned to the pGADT7 vector to express the GsTIFY10a-AD and GsTIFY10e-AD fused proteins.

For homodimerization analyses of GsTIFY10a and GsTIFY10e, the combinations pGBKT7-GsTIFY10a/pGADT7-GsTIFY10a and pGBKT7-GsTIFY10e/pGADT7-GsTIFY10e were co-transformed into yeast strain AH109. For heterodimerization analyses between GsTIFY10a and GsTIFY10e, the combinations pGBKT7-GsTIFY10a/pGADT7-GsTIFY10e and pGBKT7-GsTIFY10e/pGADT7-GsTIFY10a were co-transformed into yeast. The pGBKT7-GsTIFY10a/pGADT7 and pGBKT7-GsTIFY10e/pGADT7 combinations were used as negative controls, and the pGBKT7-AtbZIP63/pGADT7-AtbZIP63 combination was used as a positive control. The transformants were selected on SD/-Trp-Leu and SD/-Trp-Leu-His-Ade medium, respectively.

### Bimolecular fluorescence complementation (BiFc) assays

The full-length coding region of *GsTIFY10a* was PCR amplified and fused into the N-terminus of the amino-terminal half of YFP protein (YFPN), to generate the GsTIFY10a-YFPN construct. The coding regions of *GsTIFY10a* and *GsTIFY10e* were fused into the N-terminus of the carboxyl-terminal half of YFP protein (YFPC), to generate the GsTIFY10a-YFPC and GsTIFY10e-YFPC construct.

BiFc assays were conducted by transient transformation of *Arabidopsis* protoplasts prepared as described [66]. Yellow fluorescence was measured by excitation at 514 nm and emission at 527 nm, by using confocal laser-scanning microscope Leica SP2 (Leica, Wetzlar, Germany). YFP fluorescence, chlorophyll auto-fluorescence and light visions were recorded in separate channels and then merged into an overlay image.

## Supporting Information

Figure S1 **Expression patterns of the Arabidopsis and wild soybean TIFY10/11 subgroup genes under 50 mM NaHCO_3_ (pH 8.5) treatment based on the RNA-seq data.** **a**. Relative expression levels of the TIFY10/11 genes under alkaline stress. The expression levels at 0 h were considered as 1. **b**. Expression levels of the TIFY10/11 genes under alkaline stress.
(TIF)
