# Supplementary figures and images for: The Positive Regulatory Roles of the TIFY10 Proteins in Plant Responses to Alkaline Stress
Source: PLoS One. 2014 Nov 6;9(11):e111984. doi: 10.1371/journal.pone.0111984 (PMC4222965; doi:10.1371/journal.pone.0111984)

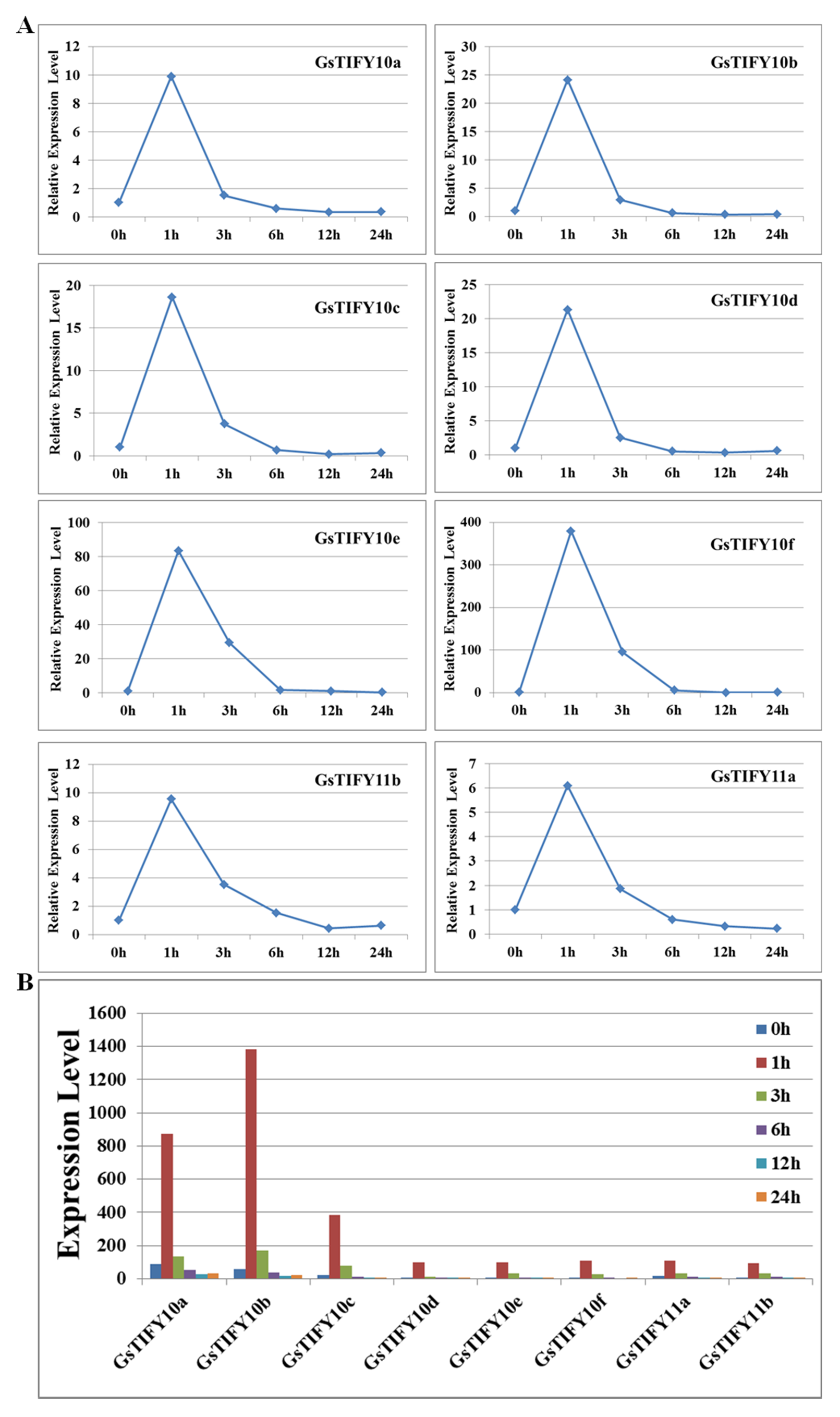

Supplement: Figure S1 — Expression patterns of the Arabidopsis and wild soybean TIFY10/11 subgroup genes under 50 mM NaHCO3 (pH 8.5) treatment based on the RNA-seq data. a. Relative expression levels of the TIFY10/11 genes under alkaline stress. The expression levels at 0 h were considered as 1. b. Expression levels of the TIFY10/11 genes under alkaline stress. (TIF) [file pone.0111984.s001.tif]
